# Joint disorder; a contributory cause to reproductive failure in beef bulls?

**DOI:** 10.1186/1751-0147-49-31

**Published:** 2007-11-05

**Authors:** Ylva Persson, Lennart Söderquist, Stina Ekman

**Affiliations:** 1 Department of Clinical Sciences, Division of Reproduction, Faculty of Veterinary Medicine, Swedish University of Agricultural Sciences (SLU), Uppsala, 750 07, Sweden; 2 Department of Biomedicine and Public Health, Division of Pathology, Pharmacology and Toxicology, Faculty of Veterinary Medicine, Swedish University of Agricultural Sciences (SLU), Uppsala, 750 07, Sweden

## Abstract

The lame sire, unsound for breeding, can cause substantial economic loss due to reduced pregnancies in the beef-producing herd.

To test the hypothesis that joint disorder is a possible cause of infertility in beef sires, right and left hind limb bones from 34 beef sires were examined postmortem to identify lesions in the femorotibial, femoropatellar (stifle), tarsocrural, talocalcaneus, and proximal intertarsal (tarsal) joints. The bulls were slaughtered during or after the breeding season due to poor fertility results. Aliquots of the cauda epididymal contents taken postmortem from 26 bulls were used for sperm morphology evaluation. As a control, hind limbs (but no semen samples) from 11 beef bulls with good fertility results were included.

Almost all infertile bulls (30/34) had lesions in at least one joint. Twenty-eight bulls (28/30, 93%) had lesions in the stifle joint, and 24 (24/28, 86%) of these were bilateral. Fourteen bulls (14/30, 47%) had lesions in the tarsal joint, and 10 (10/14, 71%) of these were bilateral. Four bulls (4/34, 12%) had no lesions, three bulls (3/34, 9%) had mild osteoarthritis (OA), 5 (5/34, 15%) moderate OA, 17 (17/34, 50%) severe OA and 5 (5/34, 15%) deformed OA. Almost all OA lesions (97%) were characterized as lesions secondary to osteochondrosis dissecans. All the bulls with satisfactory sperm morphology (n = 12/34) had joint lesions, with mostly severe or deformed bilateral lesions (83%). Consequently, the most likely cause of infertility in these 12 bulls was joint disease. Almost all control bulls (10/11) had OA lesions, but most of them were graded as mild (55%) or moderate (36%). None of the control bulls had severe lesions or deformed OA.

We suggest that joint lesions should be taken into consideration as a contributory cause of reproductive failure in beef sires without symptoms of lameness.

## Introduction

The lame herd sire, unsound for breeding, can cause substantial economic loss [1], especially when the bull is used for natural service. In recent years, Swedish farmers have become more aware of the impact of leg weakness on the fertility of the bulls. A Swedish insurance company (AGRIA) reports that many of the insured beef sires are culled because of joint problems (Ohlén, personal communication 2004). In a group of healthy, performance-tested yearling beef bulls, 97.8% had joint lesions, at slaughter, compatible with osteochondrosis (OC) [2]. Osteochondrosis [3,4], osteochondrosis dissecans (OCD) [5,6] and osteoarthritis (OA) [7] can be found in beef sires, regardless of breed.

Osteochondrosis of the articular-epiphyseal-cartilage complex (AECC) is characterized by focal disturbed enchondral ossification of the epiphyseal growth cartilage. The aetiology is not fully understood but there is strong evidence that focal failure of blood supply in the growth cartilage causes local ischemia, which in turn leads to focal necrosis of the cartilage, named OC latens with subsequent cartilage retention into the subchondral bone (OC manifesta) [8]. The disorder has been described in cattle [5], pigs [9,10], horses [11], dogs [12], man [13], poultry [14] and rat [15]. The aetiology of OC is thought to be multifactorial. Heredity, gender, growth rate, body weight, trauma, nutritional imbalance and anatomical conformation have been proposed as aetiological factors (for reviews see [16,17]). The focal necrosis (OC latens) and retention of growth cartilage (OC manifesta) with impaired ossification is sometimes followed by OCD [12]. The joint shape, growth rate and body weight have been suggested as factors influencing the local conditions of the tissue and hence the development of OC and OCD [8]. Osteochondrosis dissecans will cause a synovitis followed by a secondary OA [12,18], with synovial effusion and lameness as the main clinical symptoms. Bilateral joint lesions are common in bulls [5] and hence the lameness can be difficult to observe.

Earlier reports on musculoskeletal disorders, as a cause of infertility in bulls, have mainly focused on spondylosis of the vertebrae in dairy bulls [19]. To our knowledge, only a few reports describe the impact of hind limb disorder on the fertility of beef sires [20,21].

The aim of the present study was to test our hypothesis that hind limb joint disorder of the bull can contribute to infertility in beef herds.

## Materials and methods

### Animals

In the present study, an investigation of hind limb joints from 34 non-lame beef bulls, slaughtered due to infertility, was performed. These bulls were compared with 11 fertile beef bulls, slaughtered due to risk of inbreeding in the herd. The 34 infertile bulls were slaughtered during or after the breeding season due to a total reproductive failure. The bulls had not produced any calves during the last breeding season. All bulls had a normal mating behaviour according to the owners. Lameness had not been observed. Femorotibial, femoropatellar (stifle), tarsocrural, talocalcaneus, and proximal intertarsal (tarsal) joints from right and left hind limbs of these beef sires were examined postmortem. Semen samples were obtained by cauda epididymal dissection post mortem from 26 of the 34 bulls. The bulls were of five different breeds, Charolais (n = 15), Simmental (n = 7), Limousin (n = 6), Hereford (n = 5) and Angus (n = 1), and the mean age was 2.5 years (range 1–7 years). As controls, hind limb bones from 11 beef bulls with good fertility result were included. The control bulls were of three different breeds (Charolais (n = 6), Limousin (n = 3), Simmental (n = 2)), with a mean age of 4.5 years (range 2–9 years).

### Classification of joint lesions

The joints were disarticulated and examined macroscopically. The articular cartilage, synovial membrane/capsule, ligaments, menisci and subchondral bone were inspected for lesions such as cartilage fraying, wear lines, erosion, ulceration, osteochondrosis dissecans (OCD), cartilage retention, osteophytes and villiformation of synovial membranes. Osteochondrosis (OC) was diagnosed when cartilage retention into the subchondral bone was found and OCD when a cartilage rupture with a cartilage flap or loose osteochondral body could be seen. The lesions were recorded as unilateral or bilateral and graded as normal and with mild, moderate, severe or deformed osteoarthritis (OA).

**Figure 1:**
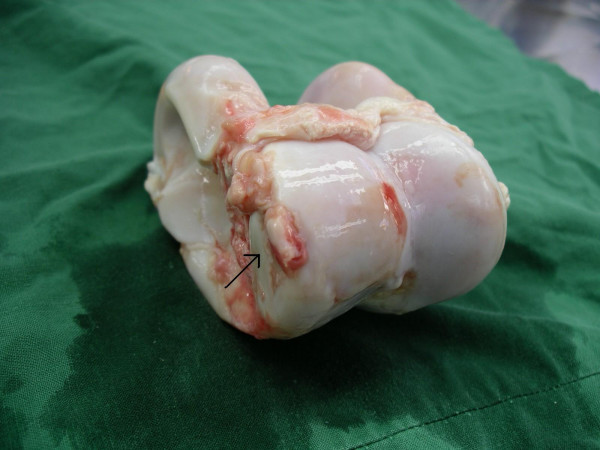
Distal trochlea of the talus. Charolais bull. Osteochondral fragmentation (OCD) of the medial condyle of the distal talus (arrow). Mild osteoarthritis (OA).

Mild OA in the femorotibial and femoropatellar joints was characterized by superficial cartilage fraying of less than 30% of the articular cartilage with single erosion <1 cm in diameter and superficial wear lines. Mild OA in the tarsocrural, talocalcaneus, and proximal intertarsal joints was characterized by single erosion or single OCD (Fig 1).

**Figure 2:**
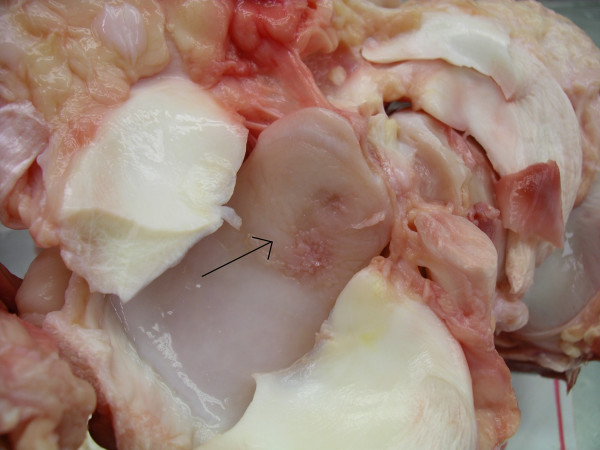
Proximal tibia. Charolais bull. Cartilage fraying and erosion (arrow) > 30% of the articular cartilage of the tibial plateau. Moderate OA.

**Figure 3:**
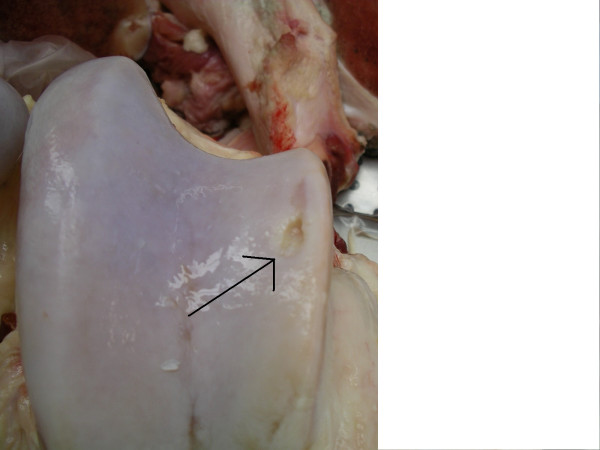
Distal femur. Simmental bull. A single ulcer < 1 cm in diameter (arrow) of the lateral trochlear ridge. Moderate OA.

**Figure 4:**
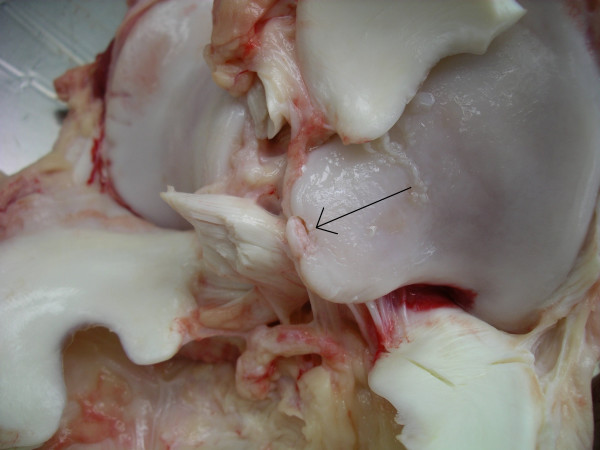
Proximal tibia. Charolais bull. An osteochondral fragmentation of the medial intercondylar eminence (arrow). Moderate OA.

Larger areas of cartilage fraying (>30% of the articular cartilage) (Fig 2), multiple erosions, single ulceration <1 cm (Fig 3), fragmentation of the intercondylar eminence of the tibia (Fig 4) and villiformation of the synovial membrane in the femorotibial and femoropatellar joints and multiple OCD and/or erosions in the tarsocrural, talocalcaneus, and proximal intertarsal joints were considered as moderate OA.

**Figure 5:**
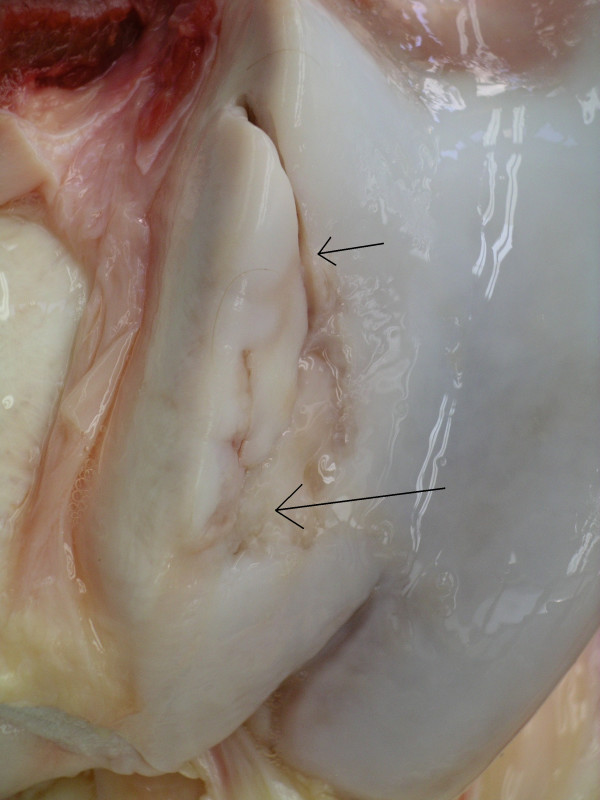
Distal femur. Charolais bull. A large osteochondral fragmentation (OCD) (short arrow) and ulceration (long arrow) of the lateral trochlear ridge. Severe OA.

**Figure 6:**
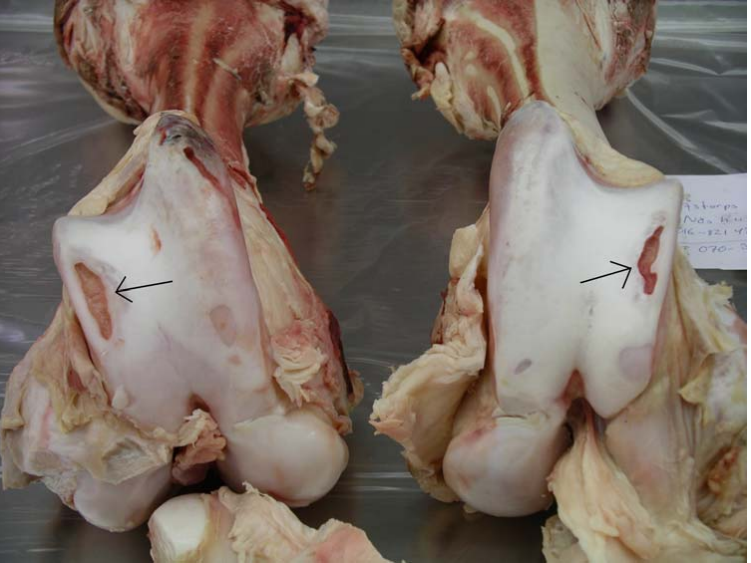
Distal femur. Hereford bull. Deep, bilateral ulcers >1 cm in length (arrows) of the articular cartilage of the lateral trochlear ridges. Severe OA.

**Figure 7:**
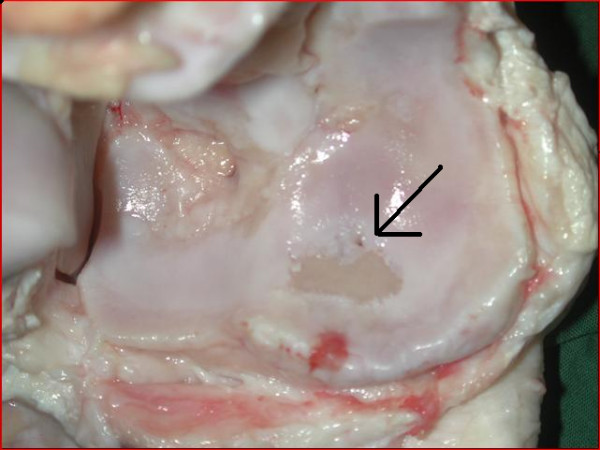
Proximal centroquartale bone. Hereford bull. Ulcer with denuded bone >0.5 cm in diameter (arrow) in the articular cartilage. Severe OA.

Severe OA in the femorotibial and femoropatellar joints was characterized by single or multiple OCD (Fig 5), multiple ulcerations and single ulcer >1 cm (Fig 6). Severe OA in the tarsocrural, talocalcaneus, and proximal intertarsal joints was characterized by ulcer with denuded bone >0.5 cm (Fig 7).

**Figure 8:**
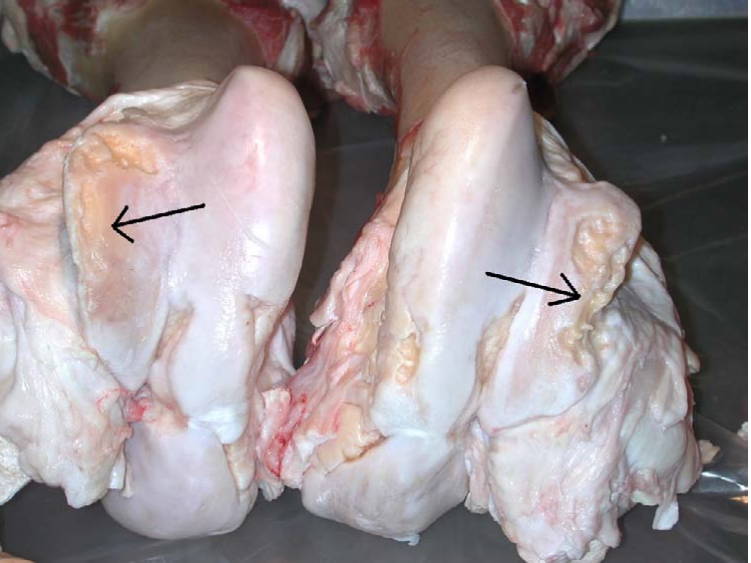
Distal femur. Charolais bull. Deep, large bilateral ulcers (arrows) with denuded bone and osteophytes in the lateral trochlear ridges. Deformed OA.

Deformed OA in both the stifle and the tarsus was characterized by severe OA with periarticular osteophytes (Fig 8).

### Sperm morphology assessments

Semen samples were collected by cauda epididymal dissection in accordance with a previous study on Swedish beef bulls [22]. The testes and epididymides from 26 of the 34 infertile bulls were weighed and examined macroscopically. Samples from the caudae epididymal fluid were used for sperm morphology examination and aliquots were used to prepare dry smears and fixed in formol saline (4–5% aqueous solution of buffered formaldehyde). Sperm head morphology was studied in smears stained with carbol fuchsine according to the method described by Williams [23] and modified by Lagerlöf [24]. Five hundred spermatozoa were counted differentially in each smear under light microscopy (x1000). The presence of proximal cytoplasmic droplets, abnormal acrosomes, detached heads, and abnormalities of the midpiece and tail were recorded in wet preparations of formal saline-fixed spermatozoa. Two hundred spermatozoa were counted in each preparation under a phase-contrast microscope (x1000). The abnormalities were classified according to the classification system by Bane [25]. Morphological abnormalities were recorded as the percentage of the total number of counted spermatozoa. To pass as a satisfactory breeder, beef bulls need to have less than 15% of any sperm abnormality (personal communication, Bane 1982). Sperm morphology was not done on control bulls.

## Results

### Bulls with impaired fertility

Locations of joint lesions in all bulls studied are displayed in Table 1. Thirty of the 34 infertile bulls (88%) had lesions in at least one joint, of which 27 (27/30; 90%) were bilateral. Twenty-three (23/30; 77%) bulls had lesions in the femorotibial joint, of which 19 (19/23; 83%) were bilateral. Twenty bulls (20/30; 67%) had lesions in the femoropatellar joint, of which 14 (14/20; 70%) were bilateral and 14 bulls (14/30; 47%) had lesions in the tarsal joint, of which 10 (10/14; 71%) were bilateral. The most common site for joint pathology was the lateral trochlear ridge of femur (20/30; 67%), followed by the plateau of proximal tibia (13/30; 43%) and the intercondylar eminence of tibia (9/30; 30%) (Table 1). Almost all of the OA lesions (29/30; 97%) were characterized as developing secondary to osteochondrosis.

**Table 1:** Different type of joint lesions with bilateral or unilateral location

		Bulls with impaired fertility		Control bulls	
Joint	Location	Bilateral	Unilateral	Bilateral	Unilateral

Femoropatellar	Lateral trochlear ridge	14	6	4	2
	Medial condyle		1		
Femorotibial	Tibial plateau	12	1	7	1
	Intercondylar eminenence	7	2	1	1
Talocalcaneus	Coracoid process of calcaneus	5	1		1
Proximal intertarsal	Lateral trochlear condyle	2	4	1	
	Medial trochlear condyle		5		
	Centroquartale bone		2		
Tarsocrural	Lateral malleolus	4	1		

Different type of joint lesions with bilateral or unilateral location for bulls with impaired fertility (n = 34) and for control bulls (n = 11).

Four of the bulls (4/34; 12%) with impaired fertility had no lesions; three bulls were classified as having mild OA (3/34; 9%), 5 moderate OA (5/34; 15%), 17 severe OA (17/34; 50%) and 5 deformed OA (5/34; 15%). See Table 2 for number of bulls with joint lesions graded from normal to deformed osteoarthritis.

**Table 2:** Number of bulls with joint lesions graded from normal to deformed osteoarthritis (OA).

Classification	Bulls with poor fertility	Bulls with unsatisfactory sperm morphology	Bulls with satisfactory sperm morphology	Control bulls
Normal	4	4		1
Mild OA	3	3		6
Moderate OA	5		2	4
Severe OA	17	4	9	
Deformed OA	5	3	1	

Number of bulls with joint lesions graded from normal to deformed osteoarthritis (OA) in bulls with impaired fertility (n = 34) and in control bulls (n = 11). Bulls with poor fertility and an unsatisfactory (n = 14) or a satisfactory (n = 12) sperm morphology are also included in the table.

### Control bulls

Ten (91%) of the 11 control bulls had OA lesions in one or both joints. Eight (8/10; 80%) of these bulls had lesions in the femorotibial joint, of which 7 (7/8; 88%) were bilateral. Six (6/10; 60%) bulls had lesions in the femoropatellar joint; of which 4 (4/6; 67%) were bilateral and only 2 bulls (2/10; 20%) had lesions also in the tarsal joint, of which 1 (50%) was bilateral. Lesions were most prevalent in the plateau of the proximal tibia (8/10; 80%), followed by the lateral trochlear ridge of femur (6/10; 60%) (Table 1). One (1/11; 9%) bull had no lesions, six (6/11; 55%) bulls had mild OA and 4 (4/11; 36%) had moderate OA. None of the control bulls had severe or deformed OA (see Table 2).

### Sperm morphology

Fourteen (54%) of the 26 infertile bulls that were assessed had unsatisfactory sperm morphology, while the rest of these 26 bulls (12) had satisfactory sperm morphology. Ten (71%) of the 14 bulls with unsatisfactory sperm morphology also had joint lesions. Of these 10; 3 had mild OA, 4 had severe OA and 3 had deformed OA. All the 12 bulls with satisfactory sperm morphology had joint lesions. Of these 12; 2 had moderate OA, 9 had severe OA and 1 had deformed OA.

## Discussion

The present results indicate that lesions compatible with osteoarthritis (OA) are common postmortem findings in beef sires, regardless of clinical history. Judged on shape and location [10] most of the OA lesions in this study were classified as being secondary to osteochondrosis (OC) with subsequent osteochondrosis dissecans (OCD), a phenomenon that is well recognized [18]. The most prevalent location of joint lesions in this study was the lateral trochlear ridge of distal femur, a predilection site for OC, also reported in previous studies, mainly on dairy bulls [26-28,6]. Most of the lesions found in the femoropatellar joint of the infertile bulls were graded as severe or deformed OA and most of these were found at the lateral trochlear ridge of distal femur.

The severe and deformed OA with denuded bone and, often secondary, synovitis can cause pain with subsequent gait asymmetries. However, bilateral lesions result in lameness from both hind limbs, which may be very difficult to observe without a lameness examination including flexion test, joint palpation and radiological examination. Hence these bulls may appear non-lame in the field. Clinical signs of lameness associated with OCD are not frequently reported in cattle [29]. Almost all bulls in the present study had bilateral, symmetrical lesions, which is similar to what has been described in a radiological study in dairy bulls [28].

The lateral ridge of the trochlea, with a proximal position in the femoropatellar joint, is not a weight bearing articular cartilage, but is under load when the bull is mounting, which can result in pain from denuded bone. We suggest that the main clinical problem arises when the bull mounts and the patella slides over the trochlea. This pain may prevent the bull from completing the mounting and therefore lead to no pregnancies in the herd.

The present findings of severe and deformed OA in most of the (22/34) infertile bulls suggest that these bulls may have difficulties completing mounting, which would explain the lack of pregnancies in the herd. The control bulls were presented with normal joints or with mild or moderate OA, probably without clinical relevance.

The bulls in this study had been culled due to infertility with no pregnancies the last year in the herd, whereas the control bulls had served satisfactory in the herd for one or more years and were sent to slaughter due to risk for inbreeding in the herd. Hence the controls were older (mean 4.5 years) than the infertile bulls (mean 2 years), which in theory would account for higher probability of articular cartilage degeneration and development of OA. However, the control bulls were classified with fewer and milder joint lesions than the infertile bulls.

All the bulls with satisfactory sperm morphology had joint lesions with mostly severe or deformed bilateral OA lesions of the femoropatellar joint (10/12). Consequently, we suggest that the most likely cause of infertility in these 12 bulls was leg weakness and not poor sperm morphology. The high incidence of sperm abnormalities found in the 14 bulls with unsatisfactory sperm morphology was probably the main cause of the infertility seen in the 7 bulls with normal joints or only mild OA. In the remaining 7 bulls however, the unsatisfactory sperm morphology could not alone explain why these bulls failed to reproduce completely. However, in this group we recorded a moderate to severe OA that together with the deteriorated sperm quality may have contributed to the total reproductive failure among these bulls. The reason for the poorer sperm quality in this group is unknown, but a negative effect of pain, caused by moderate or severe OA, on the spermatogenesis can not be excluded.

In conclusion, the present results suggest that hind limb OA can contribute to lower breeding results, probably mainly by rendering the bulls difficulties when mounting, but also by indirectly affecting the spermatogenesis negatively. Hence, joint lesions should be taken into consideration as a contributory cause of reproductive failure in beef sires with or without symptoms of lameness. Special attention should hence be paid to the bulls' hind limbs and gait when performing the bull breeding soundness evaluation.

## Authors' contributions

YP participated in the design of the study, carried out the macroscopic examination of joints, performed the analysis of the joint and sperm data and drafted the manuscript. LS participated in the design of the study and in the analysis of the sperm data and helped to draft the manuscript. SE participated in the design of the study and in the macroscopic examination and evaluation of joints and helped to draft the manuscript. All authors read and approved the final manuscript.
